# Prediction, pain, and the power of context: toward a contemporary identity for the chiropractic profession

**DOI:** 10.1186/s12998-026-00669-5

**Published:** 2026-08-10

**Authors:** Dave Newell, Solomon Segal, Giacomo Rossettini, Søren O’Neill, Tim Lothe-Raven, Lise Lothe, Steven Weiniger, Alister DuRose, Brad Beira, Keith Walker, Jorge E. Esteves, Mick Thacker

**Affiliations:** 1 AECC School of Chiropractic and Centre for Pain and Active Inference (PAIn) Research, Health Sciences University, Bournemouth, UK; 2 Department of Physiotherapy, Faculty of Sport Sciences, Universidad Europea de Madrid, Villaviciosa de Odón, Spain; 3 School of Physiotherapy, University of Verona, Verona, Italy; 4 Medical Spinal Research Unit, Lillebaelt Hospital, University of Southern Denmark (SDU), Odense, Denmark; 5 Grimstad Kiropractorklinikk, Grimstad, Norway; 6 University of Western States, Portland OR, USA; 7 Health Business School, Health Sciences University, Bournemouth, UK; 8 University of Plymouth, Plymouth, UK; 9 Escola Superior de Saúde Atlântica, Universidade Atlântica, Lisbon, Portugal; 10 RCSI University of Medicine and Health Sciences, Dublin, Ireland

**Keywords:** Predictive processing, Clinical reasoning, Pain perception, Contextual effects, Expectations, Manual therapy, Professional identity

## Abstract

**Background:**

Musculoskeletal (MSK) pain is a leading cause of disability worldwide, with low back and neck pain among the largest contributors to years lived with disability. For many people with persistent pain, mechanisms are increasingly understood as arising from altered regulation at multiple loci rather than simple ongoing tissue injury. This challenges chiropractic’s long-standing reliance on body focused explanatory models centred on correcting structural lesions or dysfunction.

**Main Body:**

Active Inference (AIF) provides a contemporary framework for understanding pain perception as an embodied, context-sensitive process of sense-making. Here, inferences concerning threat or safety are informed by information generated across multiple interacting levels (neural, physiological, psychological, behavioural, and socio-cultural), and pain emerges when threat-related inferences remain persistently weighted as trustworthy. From this perspective, manual therapy may be understood as a coherent ensemble of interacting sensory, affective, interpersonal, and contextual cues that together reshape expectations, meaning, and action. We revisit the concept of Contextually Aided Recovery (CARe), a lens that sees the therapeutic encounter as a rich space of potential contextual modulation and affordances and argue that chiropractic’s distinctive contribution lies in combining skilled manual contact with the deliberate shaping of context to support adaptive regulation.

**Conclusion:**

We propose that integrating AIF and CARe offers the chiropractic profession a coherent, evidence-aligned route toward a more contemporary identity, with implications for practice and education.

## Introduction

In this paper we open a conversation about the persistence of a reductive, body-first worldview within the chiropractic profession, one that continues to shape the rationale for chiropractic care and sustain a professional identity organised around “fixing” presumed structural or mechanical problems. We argue that the central problem is not that chiropractic has overvalued the body, but that it has often treated the person as if bodily tissues can be understood apart from experience, meaning, and context.

Historically reinforced by a Cartesian inheritance, this separation has encouraged mechanistic explanations of pain and recovery that narrow how chiropractors describe their identity, therapeutic expertise, and the nature of clinical change. Yet contemporary accounts of perception and action increasingly emphasise that human experience is not generated within the body in isolation, but emerges through inseparable dynamics spanning brain, body, and environment. From this perspective, pain is not simply a readout of tissue state, but an embodied, context-sensitive process shaped by how organisms interpret and respond to what matters for action, safety, and survival.

Active Inference (AIF) offers a coherent framework for this view. Rather than locating pain in tissues or in the brain alone, AIF understands pain as an emergent aspect of embodied sense-making, shaped by prior experience, current context, and inferences concerning threat and safety [1]. This perspective helps explain why pain may persist in the absence of clear pathology and why touch, movement, communication, and the wider therapeutic context may be central to clinical change in non-specific MSK pain [2–4]. It also allows this argument to avoid collapsing into either biomechanical reductionism or a new form of “everything is in the mind”.

Our aim is not to diminish the importance of the body or of skilled manual care, but to situate them within a broader account of patients and practitioners as integrated, self-regulating, meaning-making organisms. In what follows, we examine how inherited explanatory models continue to shape chiropractic identity and ask whether a more contemporary, embodied, and context-sensitive account of therapeutic change might offer a more coherent basis for the profession’s future development.

## Section 1: Pain and chiropractic professional identity

MSK pain represents a substantial global burden, with low back pain and neck pain consistently ranking among the largest contributors to years lived with disability worldwide [5–6]. Persistent pain makes up a disproportionate share of this burden due to high health and work-related costs and profound impacts on patients’ lives [7–9], with a significant proportion of this type of MSK pain presenting in clinical settings [10–14]. Emerging approaches to care increasingly reflect the contemporary view that persistent pain involves aberrant regulation and processing of nociceptive and interoceptive signals rather than ongoing tissue injury alone [8, 15–16].

For example, consider two patients with ostensibly similar radiographic findings of lumbar disc degeneration, normal inflammatory markers, and no spinal canal compromise. If pain were simply a reflection of structural damage, these two patients should report similar symptoms, yet in practice there are often marked differences in their pain, disability, and lived experience. Every chiropractor has seen some version of this contrast: patients with extensive degeneration who remain mobile and active, alongside others with relatively minor changes who are significantly limited. Such differences are increasingly understood within a framework that sees pain as shaped by how bodily signals are interpreted in relation to prior experience, expectations, meaning, and context [17–21].

The International Association for the Study of Pain (IASP) defines pain as ‘*an unpleasant sensory and emotional experience associated with*,* or resembling that associated with*,* actual or potential tissue damage*’ [22]. Contemporary work also distinguishes broad mechanistic categories including nociceptive, neuropathic and nociplastic mechanisms which, while overlapping and still evolving, offer a useful way of understanding why similar structural findings can generate divergent experiences [7, 23–26] (Table 1).

**Table 1:** Classification of pain mechanisms and key features

Type of pain	Definition	Examples	Key features
Nociceptive pain	Arise from activation of peripheral nociceptors by noxious stimuli.	Sprains, strains, fractures	Adaptive, sometimes proportionate to tissue injury
Neuropathic pain	Results from damage or disease affecting the somatosensory nervous system.	Radiculopathy, diabetic neuropathy, post-herpetic neuralgia	Burning, shooting, or electric-shock–like; often with sensory deficits
Nociplastic pain	Pain from altered nociceptive processing often without evidence of tissue or nerve damage.	Fibromyalgia, chronic low back pain (without structural lesion)	Disproportionate pain, hypersensitivity, influenced by multisystem inference and context

Note: While these categories are useful, ongoing research continues to uncover important additional complexities in attempts to classify nociplastic pain. For example [27–28]

For many patients, pain persists beyond expected healing timelines. Within an Active Inference framing, covered in more detail in “Section 2: The doors of perception”, this persistence can be understood as reflecting altered regulation across the whole person, with threat-related interpretations becoming disproportionately weighted within interacting cognitive, neuro-endocrine-immune, and nociceptive processes operating across multiple scales [19, 29]. In this paper, we use the term persistent pain to refer primarily to this clinical reality.

Chiropractic is consistently on the frontline of caring for persistent MSK pain. Studies indicate that a substantial proportion of patients presenting for chiropractic care have had pain for greater than three months [30], a duration consistent with the ICD-11 classification of chronic pain as a unique pain condition [13]. Yet common chiropractic explanations of pain and recovery still tend to privilege local tissue findings and structural disturbance, implicitly assuming that the musculoskeletal system is the principal driver of symptoms and improvement. We argue that this orientation sits uneasily with contemporary understanding of persistent pain, particularly its transition from acute pain to long-term disability [9, 15]. This mismatch has consequences. Patients may be told their pain reflects wear and tear, instability, or degeneration, explanations that, however well-intentioned, can and do inadvertently reinforce threat-based beliefs and protective behaviours [31–33]. Patients attending for chiropractic care often arrive already inclined toward biomechanical explanations, likely because such narratives remain common within the profession’s own discourse [34]. Importantly, such traditional somatic explanations are not merely incomplete but can become active contributors to chronicity by amplifying threat-based inference and avoidance [35, 36].

Calls to attend to context, expectancy, and non-specific effects in chiropractic are not new. For example, more than two decades ago, Jamison [37] drew attention to the role of non-specific intervention in chiropractic care. The present account should therefore be understood as building on this longer trajectory, rather than as proposing an entirely novel departure.

Understanding persistent MSK pain as a problem of *inference under uncertainty*^1^ reframes what it means to provide effective care. The task of the chiropractor here is not only to work with tissues, but to support patients in recalibrating how they interpret, predict, and respond to bodily signals within their lived context. Many experienced chiropractors already recognise this in practice, and the biopsychosocial model has become familiar across healthcare settings, albeit with varying degrees of success [18, 38–39] However, broad chiropractic discourse and practice continue to underplay beliefs, expectations, and context, reflecting persistent body-centric biomedical assumptions [40].

We argue for a more coherent account of chiropractic care that places manual therapeutic approaches within a broader and more contemporary understanding of how clinical change occurs. This is not to devalue technical skill, but to align its interpretation with emerging evidence from pain science, cognitive science, and philosophy. As such, clinical outcomes in these encounters are increasingly understood to arise from multiple mechanisms [41–42], with touch, movement, and manipulation shaped in part by the relational and affective contexts in which they are delivered [43–45].This framing presents chiropractic with an opportunity to move beyond historical narratives, but requires education that combines hands-on and reasoning skills with a strong understanding of modern pain mechanisms [46–48].

## Section 2: The doors of perception

In what follows, we occasionally use “mind” and “body” as a convenient shorthand. This is intended only for readability, not to suggest that perception or pain can be reduced to brain-bound processes. Rather, contemporary formulations of Active

**Table:** 

**Box 1. Active Inference: One of a broad family of frameworks**	
A brief clarification regarding theoretical pluralism is warranted. Predictive processing, Active Inference, and affordance-based approaches are not single, settled theories, but families of accounts that differ in significant respects. At one end of this spectrum, strongly representational and internalist readings treat the brain as inferentially secluded from the world, inferring the hidden causes of sensory input in a manner analogous to scientific hypothesis testing [49]. More moderate, action-oriented formulations retain internal generative models, while foregrounding embodiment, action, and the agent’s ongoing engagement with its environment [50]. At the other end, explicitly embodied, enactive, and ecological accounts locate inference within the dynamics of the whole organism coupled with its environment. On these accounts, affordances are understood as relational opportunities for action rather than internally represented states, and, in stronger versions, the need for internal representations is itself called into question [51–53]. These debates remain live, and we do not seek to resolve them here. Rather, we state our position plainly: throughout this paper, we adopt an embodied, enactive-ecological reading of Active Inference, acknowledge its close relationship to broader enactive and ecological traditions [31, 33, 40, 41], and use the term “affordance” in its ecological sense. Readers seeking a fuller philosophical treatment are directed to that literature	

 Inference understands perception and action as embodied, integrated, and ecological, spanning neural, immune, autonomic, endocrine, behavioural, and relational systems [19, 54–55]. From this standpoint, perception is not a passive registration of sensory input, but an active process of inference shaped by the organism’s prior experience and ongoing engagement with its world.

### Perception as inference: predictive processing

Early accounts of perception often implied that sensory systems deliver an internal picture of the world which is then “read off” by a viewer in the head. These *theatres of the mind* are intuitive because our lived experience includes a powerful sense of presence and perspective, as if someone is just behind the eyes. However, this is not how perception is generated.

The contemporary solution to perception generation, rests on early ideas that can be traced back to Helmholtz in the mid-19th century, who argued that perception is a form of unconscious inference [56]. Rather than a theatre of the mind, living organisms actively generate predictions about the causes of sensory input and refine these predictions when they are not supported by the available sensory evidence [57].

### From prediction to action: active inference

Active Inference extends predictive coding accounts by treating perception and action as inseparable. From an evolutionary perspective, organisms that can anticipate and respond adaptively to deviations from viable states are more likely to survive. This imperative is formalised in the Free Energy Principle [58], which describes how living systems tend to avoid states that are surprising, destabilising, or incompatible with continued existence, centred on those that threaten physiological or social stability (for example, being too hot, too cold, too hungry, injured, isolated, or under threat).

However, organisms do not have direct access to the world beyond the sensory signals available to them, and they are left to infer what is likely to be causing those signals. To do this, they acquire generative models of both the external world (exteroception) and the internal bodily milieu (Interoception) [59], against which incoming sensory signals can be interpreted. These models are not of the whole world, but of the structured niche the organism reliably inhabits. Importantly, they do not function as neutral representations of what is “out there” or “in here”. In many circumstances they are biased towards safety, often erring on the side of detecting threat, because the cost of missing danger may exceed the cost of a false alarm. This has obvious implications for pain and other bodily symptoms, particularly when protective inferences become overly stable or excessively precise.

Active Inference also aligns naturally with the concept of allostasis which is the idea that organisms regulate by prediction, adjusting internal and behavioural states in advance based on anticipated change, rather than merely reacting once homeostatic limits are breached [60]. Perception, in this view, is not a passive readout of sensory input.

At a simplified level, Active Inference [61] proposes that agents minimise mismatches (prediction errors) between expected and sensed states in two complementary ways:

1. by updating internal models to better accommodate new evidence (perceptual inference).
2. by acting in ways that bring about the sensory states the agent expects (active inference) [49, 62].

In practice, perceptual and active inference are deeply intertwined. Beliefs, behaviour, bodily regulation, and environmental affordances continuously shape one another.

### Priors, precision, and sensory evidence

In Active Inference, three variables are central for understanding perception and pain.

1. **Priors.** These are learned generative models about the likely causes of afferent signals. Priors exist across hierarchical levels, from low-level sensorimotor regularities to higher-level abstract narratives such as “*my spine is fragile*” or “*movement will injure me*” [63].
2. **Sensory evidence**. These are real-time signals from tissues and nerves. They are inherently noisy, ambiguous and incomplete.
3. **Precision-weighting**. This is a context-sensitive probabilistic process that determines what is trusted more in the moment, prior expectations or sensory evidence. Precision is not fixed: it is shaped by threat, safety, attention, prior learning, and social context, and it can be altered through the therapeutic encounter [64].

**Fig. 1:**
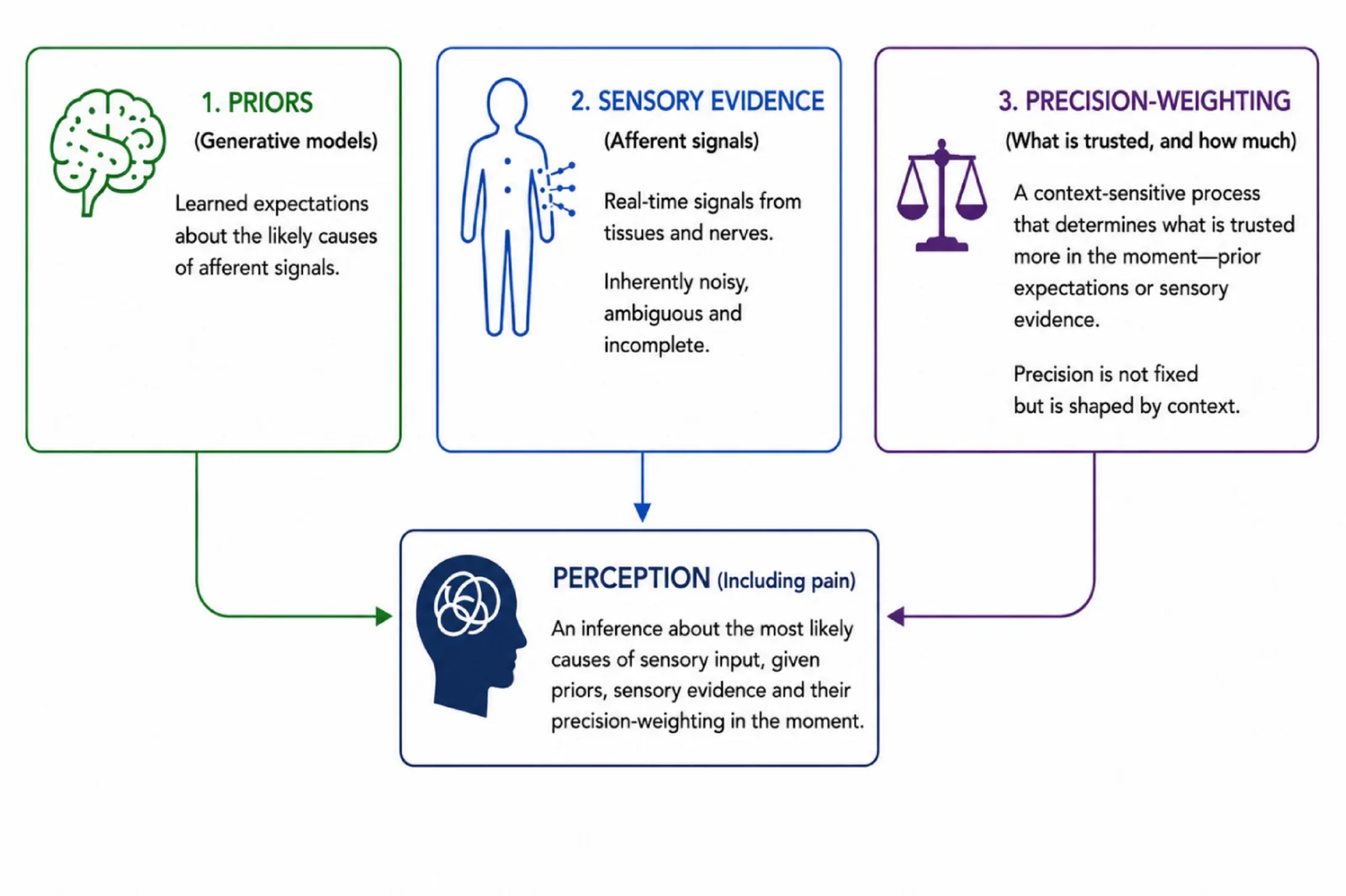
The Active Inference framework consists of 3 central mechanisms: (17). Generative models of the exteroceptive and interoceptive world (**Priors**), (22) **Sensory data** and (15) probabilistic ‘judgment’ (**Precision**) of which of these 2 information states (priors or sensory data) are most likely to best represent the world

Perception emerges from the dynamic interplay between these three elements (See Fig. 1).

For example, a patient who expects a particular movement to be harmful may experience an ordinary, non-injurious stretch as sharply painful, whereas the same movement, performed unguardedly during an absorbing task, may pass almost unnoticed. In both cases, the sensory input may be broadly similar, but the inference drawn from it differs.

In the context of pain, if high precision is assigned to higher level threat-based priors (Table 2), then ambiguous sensory signals are interpreted through the lens of high-precision threat priors and experienced as dangerous and painful [20–21] Conversely, if credible cues of safety or successful action experiences reduce the precision of threat priors and increase the weighting of benign interpretations, pain can reduce even when sensory input remains unchanged [20, 63, 65–68]. This is not because pain is imaginary, but because perception is always the result of inference under uncertainty [69].^2^

**Table 2:** Comparison of lower- and higher-level priors

Hierarchy level	Prior domain/system	Instantiated “Belief” (the system’s prediction)	Clinical example of maladaptive prior
High-Level (Most Abstract)	Cognitive/Narrative (Consciously Accessible Beliefs and Expectations)	The prediction about the future, outcomes, and identity (e.g., “This pain is permanent,” or “I am disabled.“)	Catastrophizing: A strong prior belief that pain will inevitably worsen, which increases the precision (certainty) of danger signals across all lower levels
Mid-Level	Action / Motor	The prediction about the sensory consequences of an action (e.g., “Movement will hurt me.” or “I need to protect this joint.“)	Kinesiophobia/Bracing: A prior belief that movement will cause prediction error (pain), leading to action selection that restricts normal range of motion and increases protective muscle tone
Basic	Exteroceptive / Sensory (Tactile, Proprioceptive, Vestibular)	The prediction about the causes of external sensory data (e.g., what is contacting me, where I am in space, and is it a threat?)	Visual/Vestibular Mismatch: A prior belief that head movement is dangerous, leading to prediction errors from sensory input that manifest as dizziness or persistent neck pain. Hyperalgesia/Allodynia: A belief that innocuous touch is a severe threat
Deepest (Most Fundamental)	Body-Based / Interoceptive (Autonomic, Endocrine, Immune)	The system’s fundamental set point for internal physiological integrity and allostatic safety (e.g., necessary resting muscle tone, baseline inflammation, core temperature)	Chronic Allostatic Load: Persistent high-gain sympathetic arousal (fight/flight set point) and immune arousal (a sustained inflammatory set point) reflecting a core belief of imminent, internal threat

Friston distinguishes between deep models that encode expectations over longer timescales across multiple levels of a generative hierarchy, and shallower mappings that link predictions directly to immediate sensory input. [70]

Interestingly, a growing body of work suggests that such high-level modelling integrates exteroceptive, interoceptive and proprioceptive processing into a coherent subjective self-model, informed by memory, social learning and cultural understanding that can have downstream effects on the sensorimotor aspects of embodiment [71–75].

**Table:** 

**Box 2. Embodied and multiscale Active Inference (brief clarification)**	
Although Active Inference is often introduced through “Bayesian” language, contemporary accounts emphasise that inference is a property of living systems enacted across nested levels of organisation [19]. Prediction and error correction occur not only in cortical hierarchies but through autonomic regulation, interoceptive signalling, movement, immune readiness, and social coupling. From this perspective, inference is not something the nervous system does to the body, but something the organism enacts through its embodied organisation. This matters for hands-on care. Touch is not merely a signal delivered to a passive recipient but more an enacted perturbation within a coupled person–environment system that can reshape sensory statistics, precision weighting, autonomic tone, and therapeutic alliance mediated safety. Skilled practice coordinates touch, movement, language and pacing such that changes at one level support updating at others. Kim et al. and Esteves et al. have articulated this multi scaled AIF framework previously in the context of Osteopathic care [2, 51] and Jinich-Diamant et al. [4] for touch-based interventions. This avoids both mechanistic reductionism (“correcting faults”) and cognitive reductionism (“changing beliefs”), situating chiropractic within a distributed inferential process spanning physiology, cognition, and context	

### Perception as continuous inference

Prediction and error correction are continuous and not episodes triggered by new data [76]. Generative models run throughout waking and underpin experience during dreaming, albeit in dreams, sensory input is significantly attenuated [77–78].

Most of the time, perception flows smoothly, with minor adjustments maintaining coherence. But when surprise intrudes, an unexpected sound, an unfamiliar sensation, a sudden pain, prediction errors, given appropriate precision, can interrupt the stream and compel updating or action. In this case the organism may forage for more sensory evidence, revise its expectations, or act to reduce uncertainty [79–80].

This distinction matters for persistent pain. Where pain persists, entrenched threat-related priors can dominate what is perceived, especially when afforded high precision. Low-precision sensory signals that might otherwise correct these priors fail to update them. In such cases, the system can become biased toward pain even when incoming signals from tissues are relatively benign [19, 81–82].

**Fig. 2:**
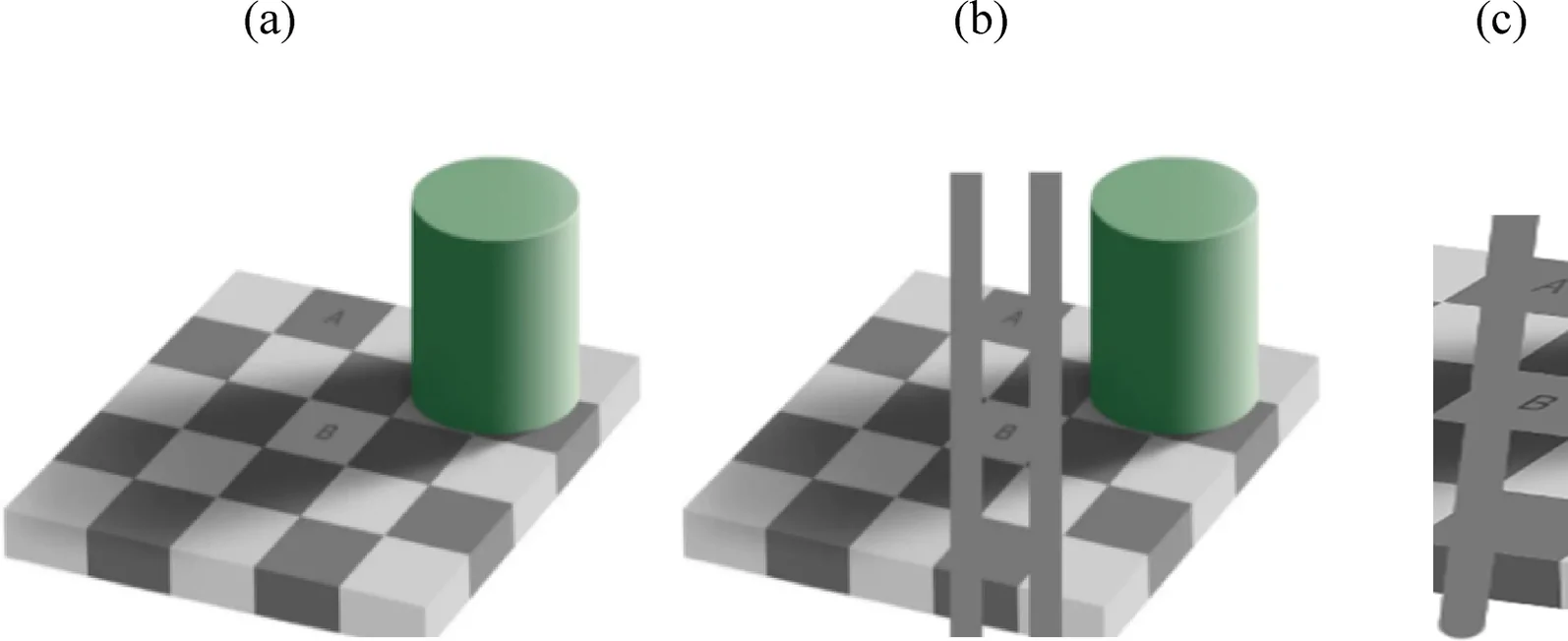
The impact of priors: the Checker Shadow Illusion [85]

A clear illustration of how prior expectations and their precision shape perception is Adelson’s checker-shadow illusion (Fig. 2). In panel a) although squares A and B reflect the same amount of light to the eye (identical luminance), the visual system does not perceive luminance directly. Instead, it infers surface lightness (how reflective each square is) by taking the surrounding context into account. Because square B is interpreted as being in shadow, the visual system compensates for the assumed reduction in illumination, leading B to appear lighter than A (see panels b and c). In this way, contextual cues such as shadows and neighbouring surfaces strongly constrain perception, overriding the raw sensory signal. Just as the visual system can “see” different shades from identical luminance when context implies different illumination, the system generating the experience of pain can “feel” different levels of pain from similar bodily signals when context and prior experience imply danger. In both cases, perception is an inferential best guess shaped by prior expectations and precision, not a direct readout of sensory input [83–84].

For chiropractic care such framing is potentially transformative. It suggests that what shapes outcomes is not confined to mechanical input but demands the cultivation of conditions in which threat-based priors can be safely revised. Hands on care, movement, explanation, pacing, and the relational quality of the encounter can all function as evidence or affordances that invite alternative interpretations and action. These contextual dynamics, and the evidence supporting them, are the focus of the Section “Section 2: The doors of perception”.

## Section 3: Contextually aided recovery

### The therapeutic encounter as a contextually rich space

The therapeutic encounter is never merely a technical intervention delivered to a passive body. It is an event saturated with meaning, expectation, and interpretation, a person caring for another person in a socially and culturally structured space. In earlier work we introduced the concept of Contextually Aided Recovery (CARe) [86] to describe the clinical encounter as replete with cues and affordances that can influence outcomes in addition to any perceived “specific” intervention delivered. We argued that these contextual dynamics are not incidental, but fundamental, and worthy of explicit recognition and deliberate curation before, during and after hands-on care.

Every clinical encounter is saturated with signals that inform high-level generative models. These include but are not limited to how the patient is greeted (both outside and inside the treatment room), the appearance of the setting, the confidence of the clinician’s voice, and the metaphors used to describe the body and pain [87]. As deeply social primates, human beings routinely look to one another as epistemic resources [88–89]. Crucially, these contextual influences shape not only what is perceived, but how patients are prepared to act. Within AIF, behaviour reflects the selection of action policies [90–92], that are judged by prior experience and present context to reduce expected uncertainty around threat and support stability. Clinical cues that signal safety, competence, and care can expand the range of actions a patient experience as possible and safe, while cues that reinforce danger or fragility can narrow behaviour toward protection and avoidance. Friston and colleagues have argued that understanding others’ behaviour is itself a form of inference, and that observation provides indispensable evidence for calibrating one’s own predictions [93]. In this sense, patients do not merely receive care but recruit the clinician and the therapeutic context as data for ongoing sense-making. From a more relational perspective, therapeutic encounters can be understood as involving partially shared generative landscapes, in which clinician and patient mutually constrain each other’s expectations and actions [94–98].

In manual therapy settings, these elements are commonly referred to as contextual factors, and a growing body of work suggests they can influence pain, function, satisfaction and recovery trajectories [99–104]. Within an Active Inference framing, contextual factors can be understood as modulators of priors and precision, i.e. they shape what the patient expects will happen and what their system is prepared to treat as credible evidence. In this light, context is not merely noise to be ignored, but part of the signal to be amplified.

### Contextual mechanisms in practice

Several domains of modifiable contextual factors have been consistently identified in the literature [100–101]. While precise effect sizes vary and remain debated in parts of the evidence base, scientific consensus supports their importance, particularly in pain where threat/safety inference and expectation are central [48, 102–104]. For chiropractic, this matters in both a positive and negative sense. Manual therapy can be enhanced when paired with credible cues of safety and agency, but it can also worsen outcomes when paired with fear-inducing explanations, catastrophic metaphors, or structurally alarming narratives [33, 36, 105]. Conversely, interventions that explicitly embody a genuine biopsychosocial orientation, without slipping into “it’s all in the mind”, can yield substantial improvements [8, 16, 106]. Table 3 summarises major domains of modifiable contextual factors, with representative examples and key references.

**Table 3:** Modifiable contextual factor domains in therapeutic encounters

Domain	Summary	Key References
Clinician–patient interactions	High-quality interactions (communication, empathy, trust, therapeutic alliance) consistently improve pain outcomes. Interaction is not just adjunctive but a core therapeutic component	[100, 107–111]
Clinician characteristics	Clinician empathy, warmth, communication style, and expectations shape patient outcomes. Positive interpersonal skills lower pain and increase satisfaction. Clinician beliefs about pain influence patient expectations and recovery	[110, 112–115]
Patient characteristics	Patients’ beliefs, expectations, and psychological traits are strong predictors of pain outcomes. High expectations, resilience, and active coping predict recovery; while catastrophizing and fear-avoidance predict poorer outcomes	[116–122]
Clinical environment	Care setting, safety perceptions, and contextual cues shape treatment outcomes. Environments signalling credibility or safety foster engagement and recovery. Natural scenes (physical or VR) can reduce pain and anxiety	[34, 123–128]
Treatment characteristics	More invasive procedures often produce stronger placebo effects. Invasiveness heightens expectations and ritualistic cues, engaging descending modulatory systems. Skilled affective touch may itself function as a salient interoceptive cue, signalling safety and supporting adaptive regulation	[2, 4, 35]

From an Active Inference perspective, contextual factors can shift priors or reweight precision toward more trustworthy evidence within the patient’s inferential system. In the language of dynamical landscapes, they can function as affordances that help move the system toward more adaptive attractors [129]. Put simply, clinical encounters can create opportunities to revise maladaptive predictions and restore flexibility in how patients engage with their bodies. Therefore, curating context is not a soft skill, nor an optional add-on to the “real work” of hands-on care. It is one of the principal levers through which chiropractic encounters shape outcomes. Contextual factors are already present in every consultation for good or bad, whether deliberately harnessed or left to chance. We contend that the ability to identify and skilfully shape these factors is not a secondary adjunct to manual skill but a core component of clinical expertise, and in many cases may be the more decisive one, because it shapes the context within which touch, explanation, and bodily change acquire meaning and therapeutic effect.

Consider, for example, preparing a patient with persistent low back pain for spinal manipulation. A formulation consistent with the present account might be:

We are going to help your back move in a way it has been protecting itself from. The pop you may hear is just pressure being released in the joint. It is a sign that movement is occurring freely, not that anything has slipped, cracked, or gone back into place. Your task is simply to allow your back to discover that it can move safely here.

In Active Inference terms, this framing reattributes a salient sensory event, namely cavitation, from a potential threat signal to a benign bodily cue. It may reduce the precision afforded to priors such as “my spine is fragile” or “something is out of place”, while positioning the clinical encounter itself as evidence against threat-based predictions [21]. On this view, manipulation operates not merely as a mechanical input, but as a structured opportunity to update higher-level predictions concerning bodily safety, movement, and capacity.

If perception is understood as active inference, movement is not merely a means of rehabilitating tissue, but a primary route through which beliefs about the body are updated. Through movement, the patient samples the body and the environment for evidence that may disconfirm threat-based predictions [67].

On this account, graded exposure can be understood as the deliberate shaping of informative prediction error. Movements are carefully adjusted in dose or intensity, so that what is experienced, such as little or no harm, differs sufficiently from what is predicted, for example “this will damage me”, to support updating, but not so markedly that the experience confirms threat and reinforces protective responses.

Several practical strategies follow from this framework. Attention may be directed externally towards a task or valued goal, rather than towards the symptomatic region. Successful performance of personally meaningful activity may be used as salient evidence of bodily capacity. Sensations likely to arise during movement may be pre-framed so that they are more readily inferred as safe. Over time, self-efficacy can be built so that confidence, rather than vigilance, becomes the patient’s default prior.

From this perspective, hands-on care and active approaches are complementary rather than competing. Touch may help to reweight precision in the moment [2, 43], while movement and exercise consolidate updated predictions through repeated lived experience [44].

Education is one of the most direct ways in which a clinician can influence a patient’s generative models. Chiropractors are often a patient’s primary source of explanation about their body and their pain, and the explanations offered in clinical encounters can therefore carry considerable weight. Pain science education, particularly the clarification that pain is a protective output rather than a direct readout of tissue damage, has been shown to reduce catastrophising and to shift unhelpful pain- and body-related conceptualisations [130]. Importantly, the constructs targeted by such education are not merely correlates of recovery. Self-efficacy, distress, and fear have been found to mediate the relationship between pain and disability [131], suggesting that changes in what a patient believes and expects are themselves part of the mechanism through which improvement occurs. Within the present framework, education works by supplying credible and coherent evidence that competes with threat-based priors and supports their revision.

A substantial part of the chiropractic profession regards the specificity of manual technique as central to clinical practice. The present framework allows this conviction to be taken seriously while also reinterpreting its basis. The precision, care, and ritual involved in delivering a specific technique may themselves function as potent contextual signals of attentiveness, expertise, and safety. These signals can shift the probabilistic weighting of incoming sensory information towards a benign, non-noxious interpretation. On this view, the therapeutic value of a precisely delivered technique need not rest solely on its mechanical specificity [132]. Active Inference offers a complementary account of why careful and precise delivery may be associated with benefit, locating part of that effect in the perceptual and predictive consequences of the encounter itself. This interpretation is consistent with evidence suggesting that the effectiveness of spinal manipulation appears largely independent of the specific application procedure [132–134]. Rather than diminishing skilled technique, this reframing helps to explain why it matters: precise, confident, and well-communicated delivery may be among the most credible safety cues a clinician can provide.

## Section 4: A roadmap for a contemporary chiropractic professional identity

### Reframing chiropractic identity

Persistent MSK pain represents one of the major contemporary health challenges. Biomechanics and tissue-based mechanisms clearly play a role in some pain presentations. However, they cannot on their own provide a sufficient account of why pain persists in the absence of clear pathology, why clinical outcomes often diverge from structural findings, or why chiropractic encounters sometimes help even when structural correction is unlikely or implausible. If chiropractic continues to rely on body-first explanatory narratives that struggle to fit these realities, it risks perpetuating a professional identity that is increasingly out of step with contemporary pain (and broader) neuroscience knowledge and with patients lived experience.

Active Inference and CARe offer a coherent alternative, not by shifting chiropractic toward an “it’s all in the mind” identity, but by clarifying why pain and recovery arise from inseparable dynamics across physiology, meaning, and action within a lived context. This matters because chiropractic already occupies a distinctive clinical space: extended time with patients, skilled hands-on care, movement guidance, and ongoing narrative exchange. Yet, without a framework that legitimises these elements and explains why they matter, the profession can default to structural rhetoric that narrows its explanatory reach and unintentionally reinforces fear, fragility beliefs, and dependence [36].

The choice, therefore, is not between a mechanical model and a psychological one. The real opportunity is to articulate chiropractic as an authentically embodied [135], contextual [28, 46–48, 86–87, 99, 102, 136–138] evidence-aligned profession: one that understands pain as inference under uncertainty [20, 65, 67, 69, 139] and views the clinical encounter as a space where threat-based priors can be stabilised, reweighted, or revised through skilled touch [2, 43] credible explanation, and curated conditions of safety [140] and agency [67].

### From rhetoric-laden holism to authentic holism

Chiropractic has long claimed to treat the *whole person* yet has often operationalised an explanatory dualism, aka a language of holistic care paired with body-dominant mechanisms. In contrast, AIF and CARe offer mechanisms and affordances [53] that dissolve this false separation. They describe one inseparable system, an embodied organism embedded in a world, generating pain and recovery through predictive regulation shaped by priors, precision, and contextual meaning.

Seen in this way, contextual skill is not an optional adjunct to chiropractic practice. It is part of the mechanism. Communication, metaphor, relational trust, safety signalling, treatment ritual, and the broader environment are not “extras”; they are active ingredients, because they alter what is inferred and what is treated as credible evidence within the patient’s system. Equally, touch and movement are not merely biomechanical inputs; they are embodied perturbations that can reshape sensorimotor confidence, autonomic tone, interoceptive inference, and the felt sense of safety, all impacting clinical outcomes in care. The distinctive potential of chiropractic lies in the coordination of these levels, not privileging one at the expense of another.

### Implications for education and public identity

If this reframed identity is to take root, chiropractic education must evolve. While many chiropractic programmes have already begun incorporating contemporary pain science and biopsychosocial perspectives, considerable heterogeneity remains internationally, and further progress is warranted. Curricula should continue to nurture specialised manual (palpation, manipulation, and mobilisation) and diagnostic skills, but must also explicitly prioritise the science of perception, action, pain, and context. A study published in 2011 [141] suggested that less than 1% of medical curricula addressed explicit education on pain mechanisms, with more recent publications continuing to identify substantial deficits that fall below recommended standards for pain education [142].

This is striking given that the vast majority of patients seek care because they are in pain [9], with chiropractors globally reporting that at least 75% of patients present with low back or neck pain [143]. Recent work has further highlighted shortcomings in pain education among professions that have historically emphasised structural approaches to care, including chiropractic, with authors concluding that *‘these professions face particular challenges in integrating contemporary pain science’* [144].

In light of the centrality of pain to future clinical practice, and the explanatory framework outlined here, chiropractic students should be taught why structural explanations are often insufficient in persistent pain, and how clinical language and metaphor can either amplify threat or support adaptive inference. They should also develop practical skills for shaping therapeutic encounters: communicating uncertainty without inducing fear, fostering agency rather than dependence, and using touch and movement as evidence of safety rather than confirmation of fragility. These skills should not be left to emerge implicitly through experience but must be explicitly embedded within modern chiropractic curricula.

This is not a call to abandon the body. The embodied self is a constitutive and indispensable dimension of the person: what we feel, how we act, and our lived sense of agency are only possible because we are embodied, and because bodily processes provide the medium through which perception, affect, and action (agency) are realised and integrated [51, 75, 145]. Rather, this is a call to abandon simplistic bodily explanations where they do not fit, and to adopt an account of the person that better reflects what contemporary pain science and neuroscience increasingly suggest, that pain is not a direct readout of tissues, but an emergent, embodied inference shaped by physiology, learning, culture, and context.

The ethical implication is clear. Chiropractic explanations are not neutral. They can either expand a patient’s possibilities for action and recovery or inadvertently entrench threat-based priors and avoidance.

### Towards a contemporary identity descriptor

If prospective students or healthcare colleagues ask what chiropractic does and why it works, how might we answer in a way consistent with contemporary evidence?

We offer the following draft descriptor as a scaffold for further development:

Chiropractors provide hands-on, person-centred care for people living with pain. Their work combines skilled physical assessment and treatment with careful attention to how each person understands, anticipates, and responds to their symptoms. Pain is shaped not only by bodily state, but also by expectation, fear, prior experience, and the context in which care occurs. Chiropractic care therefore draws on touch, movement, clear explanation, and a supportive therapeutic relationship to help patients feel safe, regain confidence in their bodies, and return to the activities that matter to them. 3

This identity descriptor does not require unverifiable claims of structural correction, nor does it reduce pain to cognition or replace existing explanations of manual care. What we attempt instead is to reposition what chiropractors do within a broader understanding of how therapeutic change occurs in the context of new evidence. Ultimately the aim of this paper is not to offer a complete account of how every element of care works, but to reframe chiropractic practice by asking a different question: what kind of system are chiropractors participating in?

### Limitations and empirical support

Active Inference is valued here not because it offers a complete or conclusively verified theory of pain, but because it provides a unifying explanatory framework [78]. Computational models of pain framed as perception–action cycles further support this position, conceptualising pain as an adaptive, though potentially maladaptive, control signal rather than a passive sensory readout [20, 68, 91]. In this respect, the framework brings perception, action, interoception, and cognition into a single account and offers a mechanistically coherent way of interpreting well-recognised clinical observations, including why pain may persist in the absence of clear pathology, why reassurance [146] and biopsychosocial approaches [8, 16] can reduce symptoms, and why therapeutic alliance [2, 100, 111, 147] and context [3, 46] can influence outcomes.

Well-documented clinical and experimental phenomena are broadly consistent with inferential accounts of pain and provide empirical support for the framework, although they should not be regarded as uniquely validating Active Inference. Phantom limb pain provides a compelling example, as pain can be experienced in a limb that is no longer present. While residual peripheral signals may contribute to its maintenance in some individuals [148], and the results of peripheral analgesia are inconsistent [149], the phenomenon is best explained by the emergence of pain from inferences about bodily state rather than from nociceptive input alone. The experience of pain reflects the interaction between prior beliefs, body representations, and available sensory evidence, with the relative influence of each determined by their precision [65]. Neuroimaging studies in fibromyalgia similarly demonstrate altered central pain processing in the absence of peripheral pathology, supporting the view that pain can arise from entrenched generative models rather than ongoing tissue damage [150, 151].

The placebo/nocebo literature provides particularly strong experimental support for inferential accounts of pain. Across laboratory [91, 117, 152] and clinical studies [118–119, 122], expectations induced by suggestion, learning, and context reliably modulate pain even when sensory input is held constant, consistent with pain reflecting the posterior of precision-weighted inference rather than a direct read out of nociception. Predictive coding and Bayesian models of placebo hypoalgesia [20, 82, 84, 153, 154] show that not only the magnitude of treatment expectations but their precision (confidence/reliability) is directly associated with the strength of placebo analgesia [155–160]. For example, in healthy volunteers, more precise treatment expectations correlate with larger placebo effects and corresponding neural signatures in pain-modulatory brainstem circuits (periaqueductal gray, rostral ventromedial medulla), consistent with precision-weighted integration of prior and sensory evidence [153, 154].

At the same time, modern formulations of the framework are relatively recent, and direct empirical tests of Active Inference in pain remain limited but are beginning to emerge [68, 161]. Critics are right to note that the framework at present may be challenging to operationalise clinically [162–164]. These concerns, while tempering any claim that AIF has already been decisively validated, do not negate its relevance as a scientifically serious and clinically applicable framework. Rather, we suggest that AIF is best understood as a provisional but coherent and parsimonious model that both synthesises what is already known from pain research [19, 165, 166] and generates testable hypotheses for future study.

Accordingly, we do not present AIF as a settled or exclusive explanation of pain. Instead, we argue that its value lies in its ability to integrate a broad range of established findings within pain science, while also offering a plausible account of how clinical encounters and contextual factors may shape pain through processes such as expectation, uncertainty reduction, affective regulation, and the modulation of inferred threat [19, 84, 167, 168]. In this sense, evidence from placebo, nocebo, and expectation studies are strongly consistent with its core claims [84, 167, 168]. On that basis, together with rapidly building empirical underpinnings [169], we believe its usefulness as a guide for contemporary, evidence-aligned professional identity is supported.

## Conclusion: the future

We suggest it is time to move beyond a body-focused dualism masquerading as holism and adopt an identity that is distinct, aligns with contemporary pain science and is ethically grounded. In an era where conservative strategies for persistent pain are urgently needed, and where human touch in healthcare is increasingly marginalised [97–98, 170], we hope to have provided a credible contemporary route toward a chiropractic identity that rediscovers its authentic and deeply integrative intentions.

## Data Availability

No, I do not have any research data outside the submitted manuscript file.

## Appendix: Glossary

**Table:**

**Attractor:** A stable region of a dynamic state space that the system tends to fall into and tend to remain within. Attractors represent habitual patterns of physiology, perception, and behaviour. In persistent pain, threat-weighted patterns (e.g., guarding, hypervigilance, pain expectation) can become attractors that are difficult to shift without credible safety signals and new experiences	**Niche:** The structured subset of the world that an organism reliably inhabits and is adapted to, including typical resources, threats, social structures, and cultural practices. A niche constrains which states are reachable and which behaviours are viable, shaping the organism’s expectations (priors) and how it regulates itself
**Active inference**: Perceptual inference **plus action**. The system not only updates beliefs to explain sensations but also selects actions that change what it senses, sampling the world in ways that reduce uncertainty and keep the organism in safe, workable states	**Perceptual inference:** Updating one’s best estimate of what is happening by combining prior expectations with incoming sensory evidence. In other words, perception is treated as a form of inference: the system “works out” the most likely causes of its sensations
**Affordances:** Opportunities for action made available by the environment (including the clinical environment). In care, cues and conditions can afford safety, exploration, and new movement strategies	**Policies (action policies):** Possible action plans the system can choose from (e.g., avoid, explore, guard, seek reassurance, relax). In active inference, policies are selected based on which actions are expected to keep the system safe and reduce uncertainty (formally minimise free energy)
**Allostasis:** Predictive regulation, adjusting physiology and behaviour in advance of expected demands to maintain stability and viability (rather than only reacting after problems arise)	**Posterior:** The system’s updated belief after it combines prior expectations with new sensory evidence. In plain terms: “What I now think is most likely, given what I expected and what I just sensed.”
**Contextual factors:** Elements of the encounter that shape expectations, meaning, and trust (e.g., language, clinician confidence, therapeutic alliance, environment, treatment ritual). They are not “extras”; they can actively shape outcomes	**Precision weighting:** A context-dependent judgement about what to trust more: prior expectations or incoming sensory signals. High precision on threat-based priors makes pain more likely; credible cues of safety can shift precision and reduce pain
**Ecological:** Perception and therefore pain emerge within a lived environment; the person’s expectations and regulation are shaped by the affordances and constraints of their social and physical world	**Prediction error:** The mismatch between what is expected and what is sensed. Prediction errors prompt either belief updating (“I was wrong”) or action (“change what I sample”) to reduce uncertainty
**Embodied:** Perception and therefore pain are generated by the whole organism (brain, body, and action) through coupled sensorimotor and physiological regulation, not by the brain alone	**Predictive processing:** The idea that the nervous system (as part of the whole organism) is constantly making best-guess predictions about sensory input and adjusting those guesses based on incoming signals. Perception is therefore shaped by expectations as well as sensation
**Free energy:** A formal measure of how “surprised” the system is by its sensory input, given its expectations or how well the organism’s generative model explains its sensory input. Living systems act and learn in ways that reduce free energy over time (making experience more predictable and manageable)	**Priors:** Expectations built from previous experience and biology. These can be low-level (sensorimotor predictions) or high-level (beliefs such as “my back is damaged” or “movement is dangerous”)
**Generative model:** A learned internal model of how the world and body usually behave. It generates predictions (“this is what I think is happening”) that shape perception and guide action	**Sense-making:** The process by which a living system actively interprets and responds to its situation considering what matters for its viability. We use the term primarily in this basic, adaptive sense, extending to participatory sense-making, meaning generated between people, when describing the therapeutic encounter; we do not use it in a purely cognitive or narrative sense
**Hands on Care:** Hands-on care, including assessment and therapeutic techniques involving contact, movement, and guided interaction	**Surprise:** How unexpected the sensory input is, given the system’s current model. Surprise itself is not directly measured; instead, organisms act to minimise expected surprise (and thus remain viable)
**Inference:** Working out the most likely cause of what you sense, given what you already expect. Inference is ongoing and evidence-based: it is how the system updates its best guess of what is happening	**Viability (viable states):** The set of bodily and behavioural states compatible with continued survival and functioning. Active inference can be understood as maintaining the system within this viable region of state space

## Abbreviations

AIF: Active Inference
CARe: Contextually aided recovery
IASP: International association for the study of pain
MSK: Musculoskeletal

## Footnotes

1
*Inference under uncertainty* is perception seen as the nervous system and body continually interpreting incomplete and noisy signals using prior experience to arrive at the most plausible ‘guess’ as to what such signals mean.

2 It is important to clarify that when we refer to “higher-level” or “abstract” priors in this manuscript, we are not using the term in the mathematically technical sense found in formal AIF treatments [138]. Some authors have suggested that priors can be instantiated at many scales of the organism (Table 2 and Box 2). Here, because our focus is clinical communication and professional identity, we use “higher-level priors” to refer to consciously accessible beliefs, expectations, and narratives that patients bring into clinical encounters. These map loosely onto higher levels of the cortical hierarchy, but our emphasis remains illustrative rather than mechanistic.

3 In the terms developed in this paper, skilled touch, movement, explanation, and the therapeutic relationship function as contextual cues and affordances. Together, they can reweight precision, revise threat-based priors, and support more adaptive patterns of inference and action across the whole person.
